# Protocol of the Development of a Core Outcome Set for Ischemic Stroke in Clinical Trials of Chinese Medicine

**DOI:** 10.1155/2020/2649843

**Published:** 2020-10-03

**Authors:** Qianwen Xie, Xueyi Deng, Jingmin Xiao, Xueyin Chen, Yihan He, Lihong Yang, Shaonan Liu, Jiaqi Lai, Yefeng Cai, Jingbo Sun, Xinfeng Guo

**Affiliations:** ^1^The Second Clinical College of Guangzhou University of Chinese Medicine, Guangzhou, China; ^2^The Second Affiliated Hospital of Guangzhou University of Chinese Medicine, Guangzhou, China; ^3^EBM & Clinical Research Service Group, Guangdong Provincial Hospital of Chinese Medicine, Guangzhou, China

## Abstract

*Background*. Ischemic stroke (IS) seriously impacts the quality of life of survivors. Chinese medicine (CM) has been developed for more than 2000 years and plays a key role in the treatment of ischemic stroke. Many Chinese medicine clinical trials have been conducted; however, the heterogeneity of outcome measurements and reporting limits implications of the findings in clinical practice and health policy development. Therefore, it is important to develop a core outcome set (COS) that should be used and reported in trials for ischemic stroke treated by Chinese medicine. This protocol describes the process of developing the IS-CM-COS. *Methods and Analysis*. The development of the COS will involve the following four steps: (1) A list of outcomes reported in the registered and published Chinese medicine trials of ischemic stroke will be extracted by conducting a systematic literature review. (2) An additional outcome list will be collected by semistructured interview to patients with ischemic stroke. (3) A two-round Delphi survey will be performed to prioritize and condense the outcomes. (4) In the consensus meeting, a final recommended COS will be developed. *Discussion*. The COS could improve the reliability and consistency of outcome reporting. We hope that this IS-CM-COS will be used in the future Chinese medicine trials for the treatment of ischemic stroke and improve research quality. *Trial Registration*. This study was registered with the Core Outcome Measures in Effectiveness Trials Initiative (http://www.comet-initiative.org/studies/details/1282).

## 1. Introduction

It is estimated that stroke is the second largest cause of death globally, with estimated global lifetime risk of 24.9% from the age of 25 years onward [1]. Ischemic stroke, the most common stroke type, is associated with high morbidity, mortality, and disability rates [2] and has a serious impact on the prognosis of patients; for example, patients with dysphagia due to ischemic stroke tend to suffer from pneumonia [3], and poststroke cognitive impairment significantly decreases the quality of life for patients [4]. Accumulating clinical evidence has shown that Chinese medicine including herbal medicine, acupuncture, and *Tuina* (Chinese massage) plays a vital role in the treatment of ischemic stroke [5–8].

There is, however, insufficient evidence on the efficacy of Chinese medicine for treating ischemic stroke due to the low quality of Chinese medicine clinical trials partly because of mismanagement of their clinical outcomes, such as different outcomes for similar clinical trials [9, 10], the use of improper instruments and time points [11, 12], lack of defining primary outcomes and reporting of adverse events [13], and incomplete reporting of outcomes [14]. These challenges result in exclusion of these clinical trials in meta-analyses [14] and therefore hindering the recommendation of potentially effective Chinese medicine interventions for ischemic stroke [15]. Further, the heterogeneity in measuring and reporting of outcomes limits the implications of the trial results in decision-making [16].

To address these problems, Chinese medicine clinicians and researchers should consider selecting, measuring, and reporting the important, relevant, and appropriate outcomes. This involves the development of a core outcome set (COS) to reach a consensus on outcomes which should be used and reported in Chinese medicine trials [17]. The use of a COS does not preclude the measurement of additional outcomes; rather, it indicates a minimum number of outcomes that should be collected, measured, and reported [17]. Typically, a COS identifies the outcomes of interest and how they should be defined and measured. The Core Outcome Measures in Effectiveness Trials (COMET) initiative has promoted the development and use of COSs. The scope of a COS refers to the specific interest area of health or healthcare that the COS is to be applied. The scope should be described in terms of the health condition, target population, and interventions that the COS is to be applicable to, thus, covering the first two elements of the Population, Intervention, Comparator, Outcomes (PICO) structure for a clinical trial. Additionally, development of a COS should involve key stakeholders including clinicians, patients, and caregivers.

There are currently four studies [18–21] on ischemic stroke in the COMET database. However, the developed COSs are only suitable for special patients (pediatric stroke) or specific interventions (angioplasty and stent-assisted angioplasty). It remains unclear whether the COSs can be used in clinical trials for adults with ischemic stroke being treated using Chinese medicine drugs or therapies.

Chinese medicine drugs or therapies are prescribed according to different Chinese medicine syndrome types. To treat patients with ischemic stroke using Chinese medicine, syndrome differentiation is necessary. Doctors should classify the patients into different Chinese medicine syndrome types before deciding the therapy remedies. The efficacy of Chinese medicine treatment depends heavily on whether the classification is done properly. Thus, the first step in developing COSs is finding out the distribution of Chinese medicine syndrome types for patients with ischemic stroke before evaluating the efficacy of Chinese medicine.

Differentiation of syndromes includes the cause, nature, and location of pathologic changes at a certain stage of the disease. Doctors analyze the clinical data regarding symptoms, physical signs, and disease history through inspection, auscultation and olfaction, interrogation, and palpation.

The patient's demographic data (age, sex, height, weight, and family history) as well as Chinese medicine clinical symptoms and signs (urine, stool, diet, sleep, sweat, energy, emotion, tongue conditions, pulse conditions, palm conditions, etc.) is collected. Not all the collected and listed signs and symptoms will be necessary for the diagnosis and evaluation of the syndrome type of ischemic stroke. Core typical features of the syndrome type of ischemic stroke will be found to help describe the typical features of patients with ischemic stroke.

It is important to determine whether outcomes regarding Chinese medicine syndromes could be included in a COS for ischemic stroke. Contrasting with outcomes in Western medicine clinical trials, outcomes regarding Chinese medicine syndromes would also be measured in the evaluation of the effectiveness of Chinese medicine treatment for ischemic stroke patients. There is a need for a COS for Chinese medicine clinical trials on ischemic stroke to achieve a consensus with experts and patients, which can be used to assess ischemic stroke and the associated Chinese medicine syndromes. This protocol presents the process of developing a COS for Chinese medicine clinical trials on ischemic stroke (IS-CM-COS). This IS-CM-COS will determine the short- or long-term outcomes that should be prioritized for measurement and reporting in future Chinese medicine clinical trials on ischemic stroke.

## 2. Methods

This study was registered with the COMET Initiative (http://www.comet-initiative.org/studies/details/1282). The research methods adhere to the Core Outcome Set Handbook [17], and this protocol is reported in alignment with the Core Outcome Set Standards for Reporting statement (COS-STAR) [22].

### 2.1. Scope of IS-CM-COS

The scope of this COS will include adults aged 18 years old and above with ischemic stroke at different stages. Its target interventions will be all types of Chinese medicine therapies (herbal medicine, acupuncture, and *Tuina* (Chinese massage)).  Table 1 shows the 11 recommendations of minimum Core Outcome Set Standards for Development (COS-STAD) to be followed when defining the scope of the COS in this protocol [22].

### 2.2. Design

**Figure 1:**
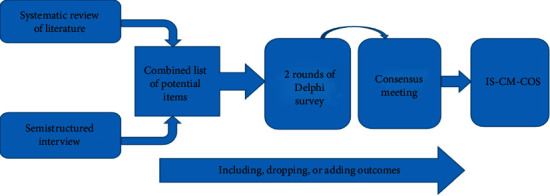
The flowchart of IS-CM-COS.

The protocol will involve four steps:

 
*Step 1*. A systematic literature review will be implemented to collect the reporting outcomes in Chinese medicine trials of ischemic stroke
 
*Step 2*. A semistructured interview will be performed to identify additional important outcomes from the patient's perspective
 
*Step 3*. A two-round Delphi survey will be conducted to prioritize and condense the outcomes
 
*Step 4*. A consensus meeting will be held to develop the IS-CM-COS (Figure 1)

### 2.3. Step 1: Systematic Literature Review

#### 2.3.1. Inclusion Criteria

We will include the following:

1. Clinical controlled trials regardless participants' allocation methods which are investigating the effectiveness and safety of Chinese medicine interventions for ischemic stroke
2. Chinese medicine interventions include any type of the Chinese medicine therapies
3. Participants are ischemic stroke patients at any stages who aged 18 years old and above

#### 2.3.2. Exclusion Criteria

We will exclude duplicates of published literature and trials that assessed the effects of treatments for comorbidities in patients with stroke (diabetes, atrial fibrillation, etc.).

#### 2.3.3. Search Strategy

We will perform a comprehensive search and identify studies published within the past two years (2017–2019) to capture outcomes reported in recent Chinese medicine clinical trials of ischemic stroke. We will search the following Chinese and English databases using MeSH terms and keywords: PubMed, Cochrane Library, Embase, the China National Knowledge Infrastructure, the Chinese Biomedical Literature Database, VIP Database for Chinese Technical Periodicals, and WANFANG Data. To ensure that more contemporary and relevant outcomes are included, we will also search clinical trial registries including ClinicalTrials.gov and the Chinese Clinical Trial Registry to collect registered protocols for randomized controlled trials investigating ischemic stroke from 2014 to 2019. The languages will be restricted to English and Chinese.

#### 2.3.4. Literature Selecting

Two reviewers (QX and XD) will independently screen the titles, abstracts, and full texts of studies. Disagreements will be resolved by consulting a third reviewer.

#### 2.3.5. Data Extraction and Analysis

Two authors (QX and XC) will independently extract data from the included studies, including authors, year of publication, study objectives, study design, sample size, characteristics of participants (age, gender, stage, duration of disease), interventions (types of therapies, duration of treatment), and outcomes (name, definition, measurement instrument/method, and measurement time). Two researchers will extract Chinese medicine syndrome types independently. The data related to Chinese medicine syndrome types will be extracted, including the syndrome name, symptoms and signs, tongue, pulse, and other syndrome information. Classification of Chinese medicine syndrome types follows the criteria of diagnosis and therapeutic effect of apoplexy and the guiding principles of clinical research on the treatment of stroke in modern CM trials. And curative effect evaluation scales such as Chinese medicine syndromes and symptom scores will be extracted. Each syndrome type explains a list of symptoms from various co-occurrence patterns. If all key manifestations of the syndrome type are present on the list, then it is regarded as well supported by the data and selected as a target for further analysis. What we are looking for is the core/key combination of symptoms (symptoms, tongue, pulse, etc.). Chinese medicine syndrome names will be standardized. Any disagreement will be discussed to achieve consensus.

A list of candidate outcomes will be developed. Outcomes with different terminology but identical or similar definition will be grouped together. On the contrary, outcomes shared one name but with different definitions will be labeled as two outcomes. Outcomes and measurements will be ranked according to their frequency in the candidate list. The remaining outcomes will be categorized into different domains [23–25]. Additionally, Chinese medicine syndrome will be added as an independent outcome domain.

### 2.4. Step 2: Semistructured Interview

The patients' opinions are important because it is the patients that experience the benefits and adverse effects of treatments. We will perform a semistructured interview to identify patient-centered outcomes. According to the research concept to theoretical saturation, we planned a convenience sample of 10 to 15 participants. According to the principles of purpose-oriented sampling, we will select stroke patients who meet our inclusion criteria from different Chinese medicine hospitals in Guangdong province [26]. These patients will mainly be from the Chronic Disease Management System of Chinese Medicine Hospitals. They either used or are currently using Chinese medicine therapy. These expert patients can better understand the prevention and prognosis of ischemic stroke and Chinese medicine therapy which will enable them to understand the purpose of our research. However, we cannot guarantee the representativeness of the sample during the sample selection. To address this issue, we will select patients varying in gender, age, disease stage, disease severity, levels of education, occupation, family income, etc. This will ensure that patients are as different as possible in the sampling process.

The questions asked during the interview will revolve around Chinese medicine therapies for ischemic stroke. After receiving clarification on the study purpose and the definition of outcomes, the patients will be asked to suggest relevant outcomes based on their experience with ischemic stroke, potentially important outcomes following Chinese medicine treatment and their reasoning based on these choices judgments. The interview outline will be pretested and updated if necessary. The outline items are as follows:

1. When was your ischemic stroke diagnosed?
2. What are the most disturbing issues for you after the stroke? Or what problems do you want to solve?
3. What are your expectations regarding the CM treatments?
4. Are there any inconvenience or shortcomings caused by the CM treatments?
5. Which outcomes are important to you? Which one is the most important?

Narrative data will be indexed and charted to produce a thematic framework. Themes will be derived from issues raised by the participants. Multiple members of the research team will discuss and agree on the outcomes that are important to patients with ischemic stroke. These patient-centered outcomes will be reviewed and added to the candidate outcome list.

### 2.5. Step 3: Two-Round Delphi Survey

#### 2.5.1. Types of Participants

Experts in ischemic stroke from different clinical departments and research institutes will be invited to form the Delphi panel, including clinicians, nurses, researchers, and statisticians. The diverse backgrounds of the panel members will ensure the comprehensiveness and practicability of the results.

There is no robust method for calculating the required sample size for a Delphi survey; therefore, we estimated the sample size based on COMET Initiative guidelines and previous studies [27–29]. Considering that no-response will occur and too many experts will lead to difficulties in investigations and consensus, the target sample size of the Delphi panel in this study is 50.

The survey consists of 2 rounds. The questionnaire will be designed based on the COS candidate items and sent to the panel members via social media apps, web links, or email. The questionnaire includes the top frequently outcome indicators. A small sample of pilot survey interviewing with neurologists, nurses, and methodology experts will be conducted before the Delphi survey. Following the suggestions from the pilot survey, we will adjust the name and classification of the outcomes and make it clear that what each outcome means and how it is defined in the questionnaire.

#### 2.5.2. Round 1-2 Delphi Surveys

We will conduct a two-round Delphi survey that will include rating scales of the importance of the outcomes and free-text fields. The questionnaires will be distributed to experts via social media apps, web links, or email and required to return within 10 days. We will use the 9-point Likert scale recommended by the Grading of Recommendations Assessment, Development, and Evaluation (GRADE) working group to obtain the importance scores of the candidate outcomes. The higher the score is, the more important the outcome is. The outcome will be categorized as limited importance (1 point to 3 points), important but not critical (4 to 6 points), and critical (7 to 9 points).

We will use Microsoft Excel 2016 or SPSS 18.0 software package to qualitatively describe the basic characteristics of the experts including educational experience, profession, professional title, and professional experience (years). We will calculate the expert positive coefficient (rate of returned questionnaire), degree of concentration for expert opinions (mean of importance score) [30], degree of coordination or consistency degree of expert opinions (variability and correlation coefficient calculated by Kendall's *W* coefficient) [31], and degree of authority of expert opinions. The degree of authority of expert opinions is calculated on the basis of experts' familiarity and judgment [32]. The experts' familiarity with the outcomes will be divided into the following 5 levels: very familiar, familiar, general, unfamiliar, and very unfamiliar. The judgment basis will be grouped into the following 4 types: theoretical basis, practical experience, peer's opinion, and intuition (choose based on feeling without the use of rational processes and even have no evidence or proof).

In the first round, the participants will receive information on the aims of the study and survey. The importance of each outcome will be scored by participants using the 9-point Likert scale. Participants can add any outcomes which are deemed important but are missing from the outcome list. Participants will be allowed to suggest whether outcomes included in the outcome list need to be modified. If new outcomes are identified or included outcomes are proposed for modification, it will be discussed before the second-round Delphi survey. The results of the first round will be shown to all the participants. If 70% or more of participants score an outcome as 7 to 9 and 30% or less of participants score it as 1 to 3 [17, 33], and the degree of authority and consistency of expert opinions are good, the outcome will be retained into the second round. The second round survey will follow the same process of the first.

### 2.6. Step 4: Consensus Meeting

In the final phase, a roundtable face-to-face consensus meeting will be held to finalize and develop the COS. All the members of the research team will attend the meeting. Experts who complete the two-round Delphi survey will be invited to attend the consensus meeting. In the meeting, we will present the results of the Delphi survey (i.e., the included and excluded outcomes). Participants will vote “yes” for including the outcome in COS, “no” for excluding the outcome from COS, and “unsure” for neutrality. The criteria of consensus are shown in Table 2 [17, 33]. In this consensus meeting, the final IS-CM-COS will consist of most core outcomes that should be applied in the clinical trials (with an expectant number of no more than 6 outcomes).

## 3. Discussion

The COS is a standardized minimum outcome set for application in clinical trials and systematic reviews, which helps translate the results into high-quality evidence. The potential bias related to the reporting of outcomes exists in Chinese medicine clinical trials due to a lack of standardization in outcome assessments [34, 35]. Varied selection of outcomes impacts on comparing and evaluating different remedies. To date, there is no COS for Chinese medicine clinical trials on ischemic stroke. We recommend that the proposed COS be adopted as a minimum set of outcomes that should be measured and reported within the given context. The development of this IS-CM-COS will improve the design and protocol of Chinese medicine trials and help them to comply with international standards.

This protocol development is consistent with the Core Outcome Set Handbook 1.0 [17] and the COS-STAR statement [22]. The outcomes will be obtained through systematic literature review and semistructured interviews. The final COS will be determined using a Delphi survey and consensus meeting.

The systematic review has an important influence on the outcome list of the COS. During the systematic review, we will search the databases for published literature and registered trials to ensure that the outcome list will include as many kinds of outcomes as possible and make sure the outcomes are commonly used at present. In the Delphi survey, the number of rounds of the Delphi survey will affect the consistency of experts' opinions. With that in mind, we designed two rounds of the Delphi survey, which is the number used by most of the studies [36–39].

The most important feature of our core outcome set is that it will include outcomes related to the Chinese medicine syndromes [40]. Although the outcomes related to the Chinese medicine syndromes are important in Chinese medicine effectiveness judgment, there are no direct evidence to prove its correlation between the Chinese medicine syndrome and the primary endpoints [41], for example, mortality and recurrence. If the outcomes related to the Chinese medicine syndrome will be included in our final IS-CM-COS, after the development of IS-CM-COS, we will conduct a cohort study to test and verify whether Chinese medicine syndrome outcomes could predict endpoints.

These detailed designs will ensure that the IS-CM-COS is able to include important outcomes for multiple stakeholders (patients, Chinese medicine physicians, Chinese medicine researchers, etc.) and make sure that our IS-CM-COS is acceptable and adopted in the future.

## Figures and Tables

**Table 1:** The COS-STAD recommendations.

Domains	No.	Methodology	Notes
*Scope specification*	1	The research or practice setting(s) in which the COS is to be applied.	The IS-CM-COS will be applied in research studies or routine clinical care.
	2	The health condition(s) covered by the COS.	The disease covered by the COS will be ischemic stroke.
	3	The population(s) covered by the COS.	The target patients will be all adults aged above 18 years old with ischemic stroke of different stages.
	4	The intervention(s) covered by the COS.	The interventions that will be covered by the COS are CM-related therapies.

*Stakeholders involved*	5	Those who will use the COS in research.	CM clinical researchers, methodologists, statistical experts, etc., will participate in the COS development.
	6	Healthcare professionals with experience of patients with the condition.	Healthcare professionals will include CM clinical experts, neurologists, and nurses.
	7	Patients with the condition or their representatives.	Patients with ischemic stroke will be included in the COS development.

*Consensus process*	8	The initial list of outcomes considered both healthcare professionals' and patients' views.	The initial list of outcomes included in the COS will be identified through a systemic literature review and interviews.
	9	A scoring process and consensus definition were described a priori.	A Delphi survey and consensus meeting will be adopted to select the outcomes.
	10	Criteria for including/dropping/adding outcomes were described a priori.	The criteria for including, dropping, or adding new outcomes will be the 9-point Likert scale recommended by the GRADE working group.
	11	Care was taken to avoid ambiguity of language used in the list of outcomes.	The language and medical terms in our COS will ensure uniformity of the outcome terms.

*Note*. Our COS will be developed following the above 11 standards. The 11 minimum reporting standards are recommended to evaluate the methodology quality of the COS study. COS: core outcome set; GRADE: Grading of Recommendations Assessment, Development and Evaluation; and CM: Chinese medicine.

**Table 2:** Criteria of consensus.

Consensus classification	Description	Criteria
Yes	Consensus that item/domain should be included in the core domain set.	Received “yes” from ≥70% of the participants and “no” and “unsure” from <15% of the participants.
No	Consensus that item/domain should not be included in the core domain set.	Received “no” and “unsure” from ≥70% of the participants and “yes” from <15% of the participants.

## Data Availability

Data and materials are available upon request from the corresponding author.

## Abbreviations

COS: Core outcome set
CM: Chinese medicine
IS: Ischemic stroke
COMET: Core Outcome Measures in Effectiveness Trials
COS-STAR: Core Outcome Set Standards for Reporting
COS-STAD: Core Outcome Set Standards for Development
GRADE: Grading of Recommendations Assessment, Development, and Evaluation.

## Additional Points

The development of the COS has started, and the systematic literature review has been completed.
